# Investigation of the Influence of Charge State and Collision Energy on Oligonucleotide Fragmentation by Tandem Mass Spectrometry

**DOI:** 10.3390/molecules28031169

**Published:** 2023-01-25

**Authors:** Christopher Gawlig, Michael Rühl

**Affiliations:** Biospring GmbH, 60386 Frankfurt am Main, Germany

**Keywords:** oligonucleotides, sequence confirmation, fragment ions, charge state, collision energy

## Abstract

Due to the increasing pharmaceutical interest of oligonucleotides, for example in antisense therapy and vaccines, their analytical characterization is of fundamental importance due to their complex structure. For this purpose, mass spectrometry is a viable tool for structural studies of nucleic acids. Structural information regarding the primary sequence of a nucleic acid can reliably be gained via tandem mass spectrometry (MSMS) fragmentation. In this work, we present the characteristic fragmentation behavior of short-chain oligonucleotides (15–35 nucleotides) with respect to the collision-induced dissociation (CID) voltage used. The relationship and influence of the length of the oligonucleotide and its charge state is also discussed. The results presented here can be helpful for estimating the required fragmentation energies of short-chain oligonucleotides and their sequencing.

## 1. Introduction

The recent years have brought more and more so called TIDES (peptides and oligonucleotides) drugs to market [1,2]. The targeted use of oligonucleotides to specifically treat genetic or rare diseases has been a chance for many patients all over the world. By now the available oligonucleotide pharmaceutical products can be classified as antisense oligonucleotides (ASOs) [3], silencing RNAs (siRNAs) [4], aptamers [5] and many more. Since the COVID-19 pandemic, mRNA vaccines and treatments have also gained popularity and have pushed the oligonucleotide field of application even further [6,7]. Advanced drug therapeutics also need advanced analytical methods to ensure the quality of these drugs. Methods already used for small molecules have found their way to the analysis of oligonucleotides. High performance liquid chromatography-(HPLC)-techniques like ion-pair reverse phase [8], ion exchange [9] or size exclusion [10] coupled to a variety of detectors are considered the standard in the industry. Mass spectrometry based techniques [11] or gel electrophoresis are used to determine the size or the molecular weight of the oligonucleotides. Mass spectrometry approaches especially are used to characterize impurities [12] from production processes or metabolites [13] from bioanalytical and pharmacokinetic measurements. Nevertheless, the determination of the exact mass using high-resolution mass spectrometers is not enough for these kinds of molecules. The sequence of the nucleotide chain must be confirmed. For oligos smaller than approximately 50 bases, tandem mass spectrometry has proven to be the method of choice [14,15,16]. Longer ribonucleic acid (RNA) molecules have been successfully sequenced by enzymatic hydrolysis approaches [17]. For mRNA, next generation sequencing and Sanger sequencing have proven to be the methods of choice but mass spectrometry has also found its way to be considered for the sequence confirmation [18] All of these techniques have one thing in common: a suitable technique to fragment the oligonucleotide has to be implemented. McLuckey et al. [14] started their investigation into the fragmentation of oligonucleotides almost 30 years ago and came up with a nomenclature of the fragments that is used since [19]. Different groups have tried different ionization methods, with electrospray ionization mass spectrometry (ESI-MS) being investigated early on and Bahr et al. [20] successfully developing a method for matrix-assisted laser desorption ionization mass spectrometry (MALDI-MS) based sequencing. In ESI-MS, the current trend focusses on pushing the limit of the maximum chain length to a range of 100 bases and above. In case of a single chromatographically and well-separated oligonucleotide analyte, it is possible to induce dissociation by a collision-induced dissociation (CID) voltage gradient and subsequently determine the appropriate voltage range without over-fragmentation of the parent ions. However, this method can lead to problems in the assignment and affiliation of fragments to their parent-ion, especially with samples that contain more than one substance and exhibit an insufficient chromatographic separation. This can make an accurate structural analysis of a single analyte considerably more difficult. An alternative would be targeted CID fragmentation of a precursor ion of the target compound. With this method, it is possible to gain a unique dissociation pattern, and thus a structural fingerprint of the analyte. Through high measurement resolution and the determination of a precursor ion, the occurrence of foreign fragments can be significantly reduced and the elucidation thus simplified considerably [14,16].

The usual instrumental measurement range of common mass spectrometers is between a mass-to-charge-ratio (*m*/*z*) of 100–2000 and thereby too small to detect the “full” mass of most oligonucleotides directly. Hence, only the multiply charged states of oligonucleotides can be measured and used as precursors for CID fragmentation. For an optimal dissociation of the target molecule without over-fragmentation, the CID voltage should be chosen at a value where the precursor ion is still visible in the spectrum (~10% base peak intensity). However, it must be considered that different fragmentation energies are required when using multiply charged precursor ions. This fact makes the estimation of a sufficient CID voltage much more problematic, since it depends on the mentioned charge state and also on the chemical composition or the length of the oligonucleotide [21]. The voltage window between insufficient fragmentation and over-fragmentation being only a few volts further complicates these estimations.

In this publication, we present a rough estimate of the required CID voltages of oligonucleotides of different lengths for sequence determination, provide an overview of the fragmentation behavior of oligonucleotides and compare the oligonucleotide (ON) length to the charge state of the precursor ion, relative to the required CID voltages. The goal was a precursor ion intensity of 10% with respect to the base peak of the fragment spectrum to ensure sufficient fragmentation and reducing the probability of over-fragmentation caused by exceedingly high voltages. The investigated oligonucleotide lengths were chosen to cover the therapeutically important range of antisense oligonucleotides of typically 15–35 nucleotides (nt) [3]. 

## 2. Results

**Figure 1:**
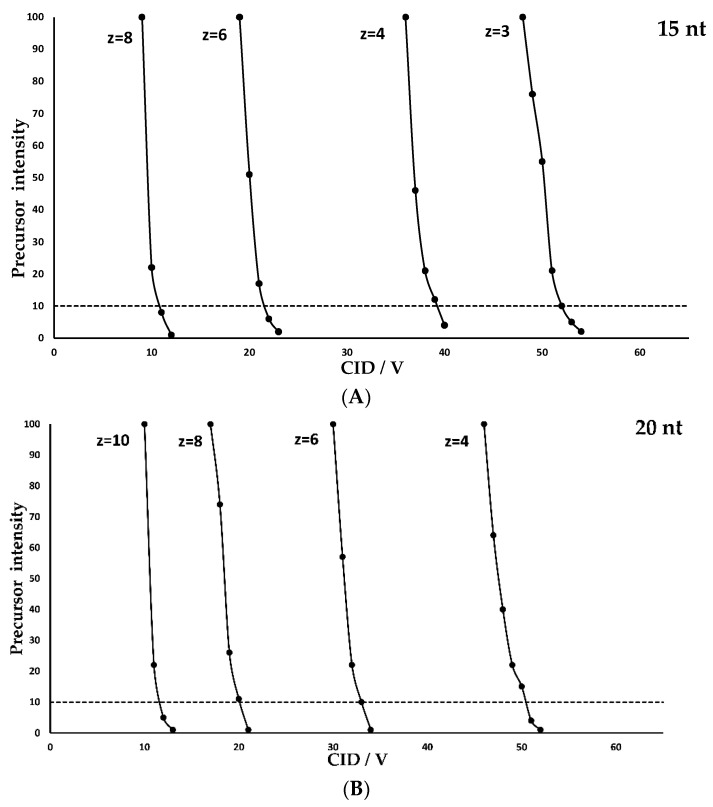

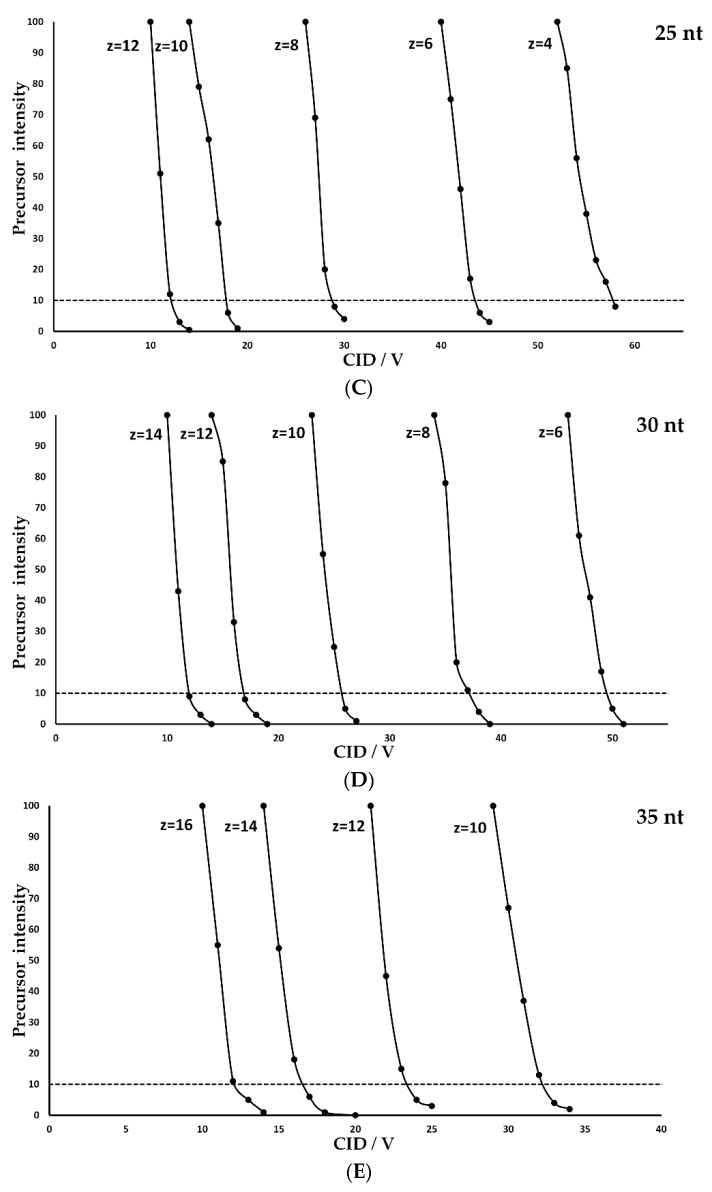
Precursor intensity at different CID fragmentation voltages in relation to charge states of poly(dT) 15 nt in (**A**), 20 nt in (**B**), 25 nt in (**C**), 30 nt in (**D**) and the 35 nt poly(T) in (**E**).

To determine a correlation of the charge state and the necessary collision energy with respect to oligonucleotide length, a desoxyribonucleic acid (DNA) polydesoxythymidine poly(dT) ladder was used for fragmentation experiments. The poly(dT) extend in steps of 5 nucleotides in the range of 15–35 nt (Table 1). In this study, the most abundant charge states in terms of intensity were used and fragmented with a wide range of CID voltages. The resulting precursor intensities were then set in relation to the voltage and are depicted in Figure 1. All poly(dT) were considered separately with respect to their charged states. It is noticeable that the voltage required to induce fragmentation depends strongly on the respective charge state. For optimal fragmentation, the precursor should be adjusted to an intensity of approximately 10% relative to the base peak to produce a sufficient fragment concentration while preventing over-fragmentation. In case of the 15 nt poly(dT) (Figure 1A) at charged state z = 3, a CID voltage of about 51 volts (V) is needed to achieve this. For higher charged states, significantly less energy is required to induce fragmentation. While 39 V are needed for a precursor ion with the charge state z = 4, only 11 V are needed for z = 8. This behavior is observed for all investigated compounds of the poly(dT) ladder. This could be due to an internal repulsion caused by a homonymous charge increase with the number of charges introduced to the molecule. At a certain level the internal forces lead to instable charge states that can only be observed utilizing supercharging agents. 

**Figure 2:**
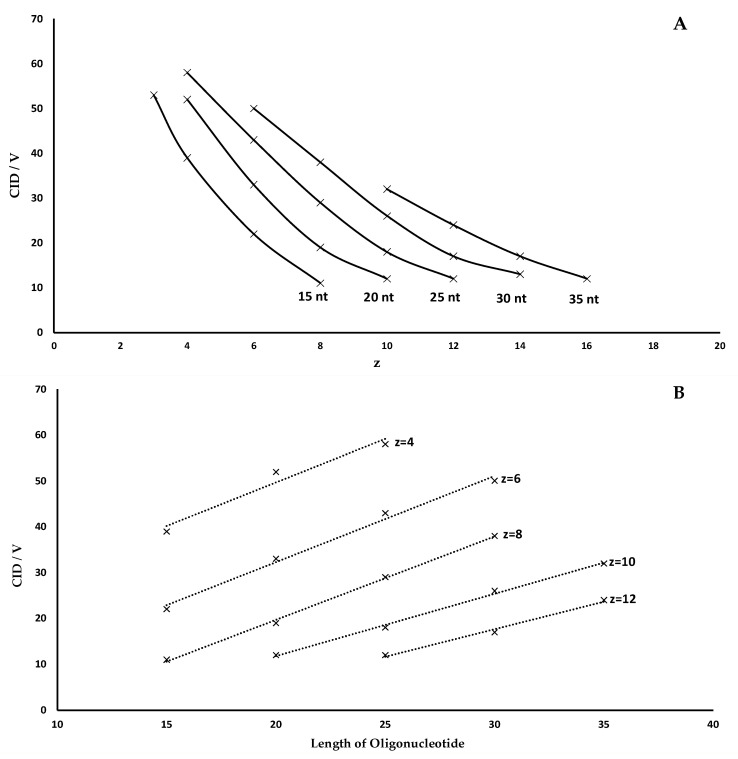
(**A**) Correlation of CID voltage and charged state. (**B**) Correlation of length to CID voltage at similar charged states.

This trend can be illustrated by correlating the charge state to the CID voltage. In Figure 2A, this is shown graphically for the different lengths of the oligonucleotides. When looking at the respective plot lines, it is noticeable that the slope decreases as the length of the oligonucleotide increases. Thus, less voltage is needed for the same degree of fragmentation between the charge states. However, this voltage window becomes much smaller at higher charge states. For the 20 nt poly(dT) (Figure 1B), the CID voltage difference (ΔCID) between z = 4 and z = 6 is about 17 V. Between z = 8 and z = 10, however, ΔCID is only 9 V. This behavior is also confirmed when observing charge states of longer oligonucleotides. For example, the ΔCID for the 35 nt poly(dT) (Figure 1E) is only 5 V between z = 12 and z = 14. Another trend is observed when considering equal charge states at different oligonucleotide lengths.

It is noticeable that, identical charge states, a significantly higher CID voltage is required for optimal fragmentation of larger oligonucleotides in comparison to smaller ones,. This observation may be caused by a dependency of the initial oligonucleotide kinetic energy to the respective charge. Therefore, higher charge states of molecules dissociate more easily in the collision cell under the influence of weak external applied energy. These influences can be the applied CID voltage as well as the applied pressure of the collision gas. The mass of the oligonucleotides correlates with the maximum number of possible charges. Hence, having a given charge state, the charges are more focused on shorter oligonucleotides compared to longer oligonucleotides and decrease the internal repulsion with increasing length.

While for 15 nt (Figure 1A), z = 8, only 11 V are needed for an optimal fragmentation, there is an increase to 20 V for 20 nt (Figure 1B), z = 8, and 38 V for 30 nt (Figure 1D), z = 8. This correlation is shown graphically in Figure 2B. Utilizing this graphic plot, it is possible to estimate the fragmentation voltages based on the length of the oligonucleotide, which also applies to heteromeric built DNA. However, it should be noted that such an estimation is only accurate for similar molecules. This means that there are no analogous CID fragmentation voltages between, i.e., DNA and RNA. Stronger modifications, such as thiolation, also lead to significant deviations in the fragmentation voltage. However, these deviations only refer to the CID voltage. The characteristics of fragmentation described in terms of charge states and oligonucleotide length remain the same and can be quickly adapted for different kind of molecules. 

**Figure 3:**
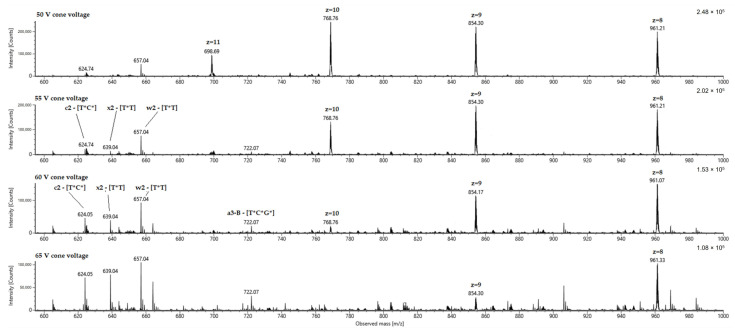
Fragmentation of a 24 nt fully thiolated oligonucleotide via increase of cone voltage. Multiple charged state signals are highlighted and fragments labelled according the McLuckey et al. [19].

In Figure 3, this is illustrated by the fragmentation of a fully thiolated 24 nt oligonucleotide (Table 1, Materials and Methods). For illustration purposes, instead of using a precursor, all charge states were fragmented by gradually increasing the cone voltage. In this case, analogous to our fragmentation characteristics described earlier, a comparison of the spectra shows that high charge states require a low CID voltage and thus fragment stepwise as the voltage is increased. Figure 3 also shows the expected fragments of 2–3 nucleotides of the 3’ and 5’ ends of the oligonucleotide in the 600–700 *m*/*z* range. These are labeled according to the nomenclature of oligonucleotide fragmentations defined by McLuckey et al. [19]. For such compounds, test measurements must also be performed in analogy to Figure 2A,B in order to subsequently estimate the required voltages for varying lengths and charge states via linear equations. However, it should also be noted that the device-specific characteristics and settings also result in significantly different voltages. For example, the pressure of the collision cell is an important constant for reproducible voltage values. For this reason, the data we publish cannot be applied directly to other systems but must be collected specifically through individual test measurements on respective devices.

Despite this, the relationship between oligonucleotide length, CID voltage and charge state, as displayed in Figure 1 and Figure 2, remains the same. Based on initial experimental measurements of the optimal CID voltage of individual charge states (Figure 1), simple estimates can be made for oligonucleotides with different lengths using the linearity shown in Figure 2B. These relationships allow the user to make reliable estimates for additional charge states or oligonucleotide lengths, independent of device-specific characteristics.

## 3. Materials and Methods

### 3.1. LC-MS/MS Instrumentation

All measurements with the poly(dT) ladder were acquired on an ACQUITY UPLC I-Class system (Waters Corporation, Milford, MA, USA) coupled with a VION IMS qTOF mass spectrometer (Waters Corporation, Milford, MA, USA), measurements with the 24 nt thiolated oligonucleotide were acquired on a BioAccord LC-MS System with ACQUITY Premier (Waters Corporation, Milford, MA, USA). Source parameters were set as: 100 V source offset, 120 °C source temperature, 400 °C desolvation temperature, 800 L/h (L/h) desolvation gas flow and 50 L/h cone gas flow. The scan range of full MS was set to *m*/*z* 500.00–2000.00 at a scan rate of 1 Hz (1 s^−1^). Data acquisition and processing were performed with UNIFI^TM^ (1.9.13.9 and 3.0.0.15). Calibration of MS data was achieved throughconstant infusion of leucine enkephalin calibration solution by Waters (SKU: 186006013) at a flow rate of 10 µL/min. 

### 3.2. Chemicals and Reagents 

All chemicals used in this publication were provided by commercial distributers. Methanol (LC-MS-grade, ≥99.97%) and Water (LC-MS-grade), used as main eluent components, were bought from Merck KGaA (Darmstadt, Germany). Eluent chemicals 1,1,1,3,3,3,-hexafluoro-isopropan-2-ole (HFIP) (≥99.8%) and triethylamine (TEA) (≥99.97%) were provided by Biosolve B.V. (Valkenswaard, Netherlands) and Merck KGaA respectively. All poly(dT) oligonucleotide samples stem from the MassPREP Oligonucleotide Standard and the analytical column ACQUITY Premier BEH C18 with 1.7 µm particle size, 2.1 mm inner diameter and 50 mm length purchased from Waters Cooperation (SKU: 186004135; Table 1; Milford, MA, USA). The 24 nt thiolated oligonucleotide was synthesized by the Biospring GmbH (Frankfurt, Germany) in house by solid phase synthesis according to Beaucage et al. [22]. Oligonucleotide analysis samples were prepared with Ambion^TM^ Nuclease-Free Water by Thermo Fisher Scientific (Dreieich, Germany). 

### 3.3. Sample Preparation 

MassPREP includes approximately equimolar amounts of 15, 20, 25, 30 and 35 nucleotides long oligodeoxythimidines poly(dT) with 1 nmole each. The oligonucleotides were solved in 100 µL of nuclease free water to a concentration of 50 µM. The fully thiolated 24 nt oligonucleotide was dissolved to a final concentration of 0.5 mg/mL (65 µM). 

### 3.4. LC-MS/MS Measurement 

The LC flow rate was set to 0.3 mL/min. Eluent A consists of a buffer system with 7 mM TEA and 80 mM HFIP solution in water. Eluent B consists of a mixture of 50% Eluent A and 50% methanol. The injection volume was set to 2 µL with the column temperature at 60 °C. Eluent B elution gradient was set as follows: 0–0.20 min (min) at 10%, 0.20–2.00 min from 10 to 100%, 2.00 to 2.30 min at 100%, from 2.30–3.00 min from 100% to 10%. All ESI-MS measurements on the VION IMS qTOF mass spectrometer were performed in negative ion mode, the ion source key parameters used were as follows: 2 kV capillary voltage, 60 V cone voltage and an offset voltage of 100 V. The source temperature was set to 120 °C with a desolvation temperature of 400 °C. The cone gas flow was set to 50 L/h and the desolvation gas flow to 800 L/h. All MSMS measurements with the BioAccord LC-MS system with ACQUITY Premier were performed via cone voltage variation between 50 and 100 V. The capillary voltage was set to 800 V with a desolvation temperature of 550 °C. All five oligonucleotides of the poly(dT) premixed standard (MassPREP Oligonucleotide Standard, see Section 3.3) were measured within one experiment on the VION IMS qTOF (Waters Cooperation, Milford, MA, USA). The collision energy was varied from 10 V to 70 V in 1 V steps and the corresponding precursor *m*/*z* values were varied to choose the corresponding charge states of the different oligonucleotides. For the experiments of the fully thiolated 24 nt oligonucleotide, the cone voltage of the BioAccord ion source was varied in a range of 50 to 65 V in 5 V steps. All experiments were performed in triplicates with independently prepared solutions on the same instrument. 

## 4. Conclusions

The MSMS experiments described in this work provide an overview of the characteristic fragmentation properties of oligonucleotides (15–35 nt). The DNA ladder investigated in this work made it possible to visualize the relationship between the charge states of an oligonucleotide and the CID voltage required for fragmentation. These relationships can be summarized as follows:

(I) The optimal CID voltage required for the fragmentation of an oligonucleotide carrying a specific charge state increases linearly with oligonucleotide length. This relationship is shown graphically in Figure 2 as described for peptides in various publications and for oligonucleotides by Ickert et al. [21] A more robust fragmentation is observed at lower charge states with higher individual fragmentation energy, while higher charge states need less energy for successful fragmentation but bear the risk of internal fragmentation, an undesired effect in sequence confirmation or multiple reaction monitoring (MRM) measurements. Additionally, high charge states have a smaller fragmentation energy window and are therefore more susceptible to variations in the fragmentation voltage, which is important for a successful method validation.
(II) For a given oligonucleotide length, high charge states require lower collision energies for an optimal fragmentation while low charge states require higher collision energies.

With the help of these correlations, it is possible to achieve an optimal fragmentation by performing preliminary experimental tests. This is of great importance in sequence confirmation as one of the most important analytical procedures for oligonucleotide pharmaceuticals. If the collision energy is set too low, no sufficient fragmentation and therefore no full sequence coverage can be obtained. Additionally, too high a collision energy can cause over-fragmentation of the oligonucleotide (internal fragmentation, fragmentation of fragments etc.) and prevent full sequence coverage as well.

By collecting individual fragmentation data of similar charged states of oligonucleotides with varying lengths, it is possible to give an estimate of the required CID voltages for MSMS experiments of oligonucleotides, analogous to the linearity shown in Figure 2B. It should be noted that such data must always be collected specifically for various instruments and that a constant pressure in the collision cell of the mass spectrometer is crucial for reproducible results. The general aspect of this work could be extended to a well characterized adjuvant 24 nt thiolated oligonucleotide. Altogether, it was possible to show the importance of the charge state and fragmentation energy choice for successful fragmentation for both academic and regulated purposes.

## Figures and Tables

**Table 1:** Sequence and mass of used oligonucleotides.

Oligonucleotide	Sequence	m/Da
15 nt	TTT TTT TTT TTT TTT	4578.70
20 nt	TTT TTT TTT TTT TTT TTT	6098.93
25 nt	TTT TTT TTT TTT TTT TTT TTT	7619.16
30 nt	TTT TTT TTT TTT TTT TTT TTT TTT	9139.39
35 nt	TTT TTT TTT TTT TTT TTT TTT TTT TTT	10,659.62
24 nt	T*C*G *T*C*G *T*T*T *T*G*T *C*G*T *T*T*T *G*T*C *G*T*T	7696.69

* phosphorothiolate linkage.

## Data Availability

The data presented in this study are available on request from the corresponding author. The data are not publicly available due to company policies.

## Sample Availability

Samples of the compounds are not available from authors.

## References

[1] Al Musaimi O., Al Shaer D., Albericio F., De la Torre B.G. (2021). 2020 FDA TIDES (Peptides and Oligonucleotides) Harvest. Pharmaceuticals.

[2] Al Shaer D., Al Musaimi O., Albericio F., De la Torre B.G. (2022). 2021 FDA TIDES (Peptides and Oligonucleotides) Harvest. Pharmaceuticals.

[3] Dhuri K., Bechtold C., Quijano E., Pham H., Gupta A., Vikram A., Bahal R. (2020). Antisense Oligonucleotides: An Emerging Area in Drug Discovery and Development. J. Clin. Med..

[4] Rossi J.J., Rossi D.J. (2021). SiRNA Drugs: Here to Stay. Mol. Ther..

[5] Ni S., Zhuo Z., Pan Y., Yu Y., Li F., Liu J., Wang L., Wu X., Li D., Wan Y. (2021). Recent Progress in Aptamer Discoveries and Modifications for Therapeutic Applications. ACS Appl. Mater. Interfaces.

[6] Pardi N., Hogan M.J., Porter F.W., Weissman D. (2018). MRNA Vaccines—A New Era in Vaccinology. Nat. Rev. Drug Discov..

[7] Park J.W., Lagniton P.N.P., Liu Y., Xu R.-H. (2021). MRNA Vaccines for COVID-19: What, Why and How. Int. J. Biol. Sci..

[8] Gilar M. (2001). Analysis and Purification of Synthetic Oligonucleotides by Reversed-Phase High-Performance Liquid Chromatography with Photodiode Array and Mass Spectrometry Detection. Anal. Biochem..

[9] Cook K., Thayer J. (2011). Advantages of Ion-Exchange Chromatography for Oligonucleotide Analysis. Bioanalysis.

[10] Shimoyama A., Fujisaka A., Obika S. (2017). Evaluation of Size-Exclusion Chromatography for the Analysis of Phosphorothioate Oligonucleotides. J. Pharm. Biomed. Anal..

[11] Sutton J.M., Guimaraes G.J., Annavarapu V., Van Dongen W.D., Bartlett M.G. (2020). Current State of Oligonucleotide Characterization Using Liquid Chromatography–Mass Spectrometry: Insight into Critical Issues. J. Am. Soc. Mass Spectrom..

[12] Pourshahian S. (2021). Therapeutic Oligonucleotides, Impurities, Degradants, and Their Characterization by Mass Spectrometry. Mass Spectrom. Rev..

[13] Lin Z.J., Li W., Dai G. (2007). Application of LC–MS for Quantitative Analysis and Metabolite Identification of Therapeutic Oligonucleotides. J. Pharm. Biomed. Anal..

[14] Mcluckey S.A., Berkel G.J.V., Glish G.L. (1992). Tandem Mass Spectrometry of Small, Multiply Charged Oligonucleotides. J. Am. Soc. Mass Spectrom..

[15] McLuckey S.A., Habibi-Goudarzi S. (1994). Ion Trap Tandem Mass Spectrometry Applied to Small Multiply Charged Oligonucleotides with a Modified Base. J. Am. Soc. Mass Spectrom..

[16] Abdullah A.M., Sommers C., Hawes J., Rodriguez J.D., Yang K. (2022). Tandem Mass Spectrometric Sequence Characterization of Synthetic Thymidine-Rich Oligonucleotides. J. Mass Spectrom..

[17] Goyon A., Scott B., Kurita K., Crittenden C.M., Shaw D., Lin A., Yehl P., Zhang K. (2021). Full Sequencing of CRISPR/Cas9 Single Guide RNA (SgRNA) via Parallel Ribonuclease Digestions and Hydrophilic Interaction Liquid Chromatography–High-Resolution Mass Spectrometry Analysis. Anal. Chem..

[18] Vanhinsbergh C.J., Criscuolo A., Sutton J.N., Murphy K., Williamson A.J.K., Cook K., Dickman M.J. (2022). Characterization and Sequence Mapping of Large RNA and MRNA Therapeutics Using Mass Spectrometry. Anal. Chem..

[19] Wu J., McLuckey S.A. (2004). Gas-Phase Fragmentation of Oligonucleotide Ions. Int. J. Mass Spectrom..

[20] Bahr U., Aygün H., Karas M. (2009). Sequencing of Single and Double Stranded RNA Oligonucleotides by Acid Hydrolysis and MALDI Mass Spectrometry. Anal. Chem..

[21] Ickert S., Schwaar T., Springer A., Grabarics M., Riedel J., Beck S., Pagel K., Linscheid M.W. (2019). Comparison of the Fragmentation Behavior of DNA and LNA Single Strands and Duplexes. J. Mass Spectrom..

[22] Beaucage S.L., Caruthers M.H. (1981). Deoxynucleoside Phosphoramidites—A New Class of Key Intermediates for Deoxypolynucleotide Synthesis. Tetrahedron Lett..

